# Analysis of Physical Demands in Four Tug of War World Indoor Championships (2010–2016)

**DOI:** 10.3390/ijerph19084871

**Published:** 2022-04-17

**Authors:** Ruth Cayero, Asier Zubillaga, Valentín Rocandio, Arkaitz Castañeda-Babarro, Ignacio Refoyo, Julio Calleja-González, Inmaculada Martínez de Aldama

**Affiliations:** 1 Department of Physical Education and Sports, Faculty of Education and Sport, University of the Basque Country, (UPV/EHU), 01007 Vitoria-Gasteiz, Spain; ruth.cayero@ehu.eus (R.C.); asier.zubillaga@ehu.eus (A.Z.); valentin.rocandio@ehu.eus (V.R.); julio.calleja@ehu.eus (J.C.-G.); inmaculada.martinezdealdama@ehu.eus (I.M.d.A.); 2 Health, Physical Activity and Sports Science Laboratory, Department of Physical Activity and Sports, Faculty of Psychology and Education, University of Deusto, 48007 Bilbao, Spain; 3 Sports Training Research Group, Universidad Politécnica de Madrid, 28040 Madrid, Spain

**Keywords:** tug of war, competition, World Indoor Championships, pull duration, gender, weights

## Abstract

Background: Tug-of-war (TOW) is one of the oldest sports in current existence and is an internationally played activity that includes professional and amateur sport athletes, played according to the rules laid out by the Tug of War International Federation (TWIF). In this type of competition, the two teams of eight members each pull against one another on a rope. The team that pulls the opposing team towards a centerline for a distance of 4 m in two pulls out of three is considered the winning team in an international competition. To the best of the authors’ knowledge, no previous scientific articles have described data during a follow-up period of Championships in TOW. Therefore, the main aim of this study was to analyze the duration of the matches, differentiating between the first (T1) and second pull (T2) of each match. Methods: The pulls were compared in the qualification point phase and the final phase, as well as in each weight category. In addition, any differences between the statistics for each of the four World Championships studied were analyzed. Finally, the total volume of pulls in each weight category was studied, both in the open club competition and in the national team competition. Data were collected from four World Indoor Championships (2010–2016). A total of 1862 matches were registered (3724 pulls), differentiating the first (T1) and second (T2) pull. The data were expressed as means (M) ± standard deviations (SD). Results: (1) The second pull is shorter than the first: T1 < T2, *p* < 0.001, (Es = 0.452; small effect); (2) T1 and T2 times were longer in the final phase than the qualifying phase: T1 phase2 > T1 phase1, *p* < 0.05 (ES = 0.469; small effect) and T2 phase2 > T2 phase1, (*p* < 0.05), (ES = 0.486; small effect); (3) there are statistical differences (*p* < 0.005) in T1 and T2 at all weights, except for T1 at 500 kg and T2 at 640 kg; (4) the comparison among World Championships does not present significant changes in the duration of the pulls; (5) however, in females, significant differences (*p* < 0.05) between the 2010 World Championships and the remainder in T1 are observed; (6) the volumes that we should take into account when designing the preparation should be those obtained when 11 teams compete against each other, considering the maximum volume that we could find in the competition. Conclusion: This first aspect of the reality of TOW indoor competitions, specifically the duration of the matches, leads to a better understanding of the demands of the sport and the type of effort required. These ideas may help coaches in the design of training plans, maximizing their specificity and their effectiveness.

## 1. Introduction

Tug-of-war (TOW) is an internationally played activity that includes professional and amateur sport athletes [1,2]. TOW is one of the oldest sports in existence, and towards the end of 19th century, it became an organized sport in different parts of Europe; it was also included in the Olympic Games program held from 1900 to 1902 [1,3]. In 1960, the TOW International Federation (TWIF) was founded. The TWIF is a member of the World Games Association, and the sport of TOW has been part of the World Games since 1981; it was included for the first time in the World Games held in the USA [1]. Every two years, TWIF organizes the World Championships, where the best TOW athletes compete. In international TOW competitions, both outdoor and indoor categories are recognized [4].

According to the rules in TOW, two teams of “pullers” participate in applying enormous contra-directional forces on the pulling rope [2], with eight members in each team pulling against one another on a rope [1,4]. The team that pulls the opposing team towards a centerline for a distance of 4 m in two pulls out of three is the winning team [5]. Originally, two sub-types of competition were used: knockout and points [3]. First of all, the teams pull in the qualifying point competition. In that way, each team may win, lose or tie [4]. Finally, the top-ranked teams after the preliminary competition enter into the semi-finals (when there is a tie, a third pull is made). The same criteria apply in the finals and in the fight for the bronze medal [4]. In the World Championships the following weights for senior pullers are used: male categories, 560–600–640–680–700 k; and in the female category, 540 k and 500 k. Another category began to be used in 2012, which is the mixed category (four women and four men). If there are 12 or more teams in each weight class, the qualifying point competition will be divided into groups [4], so the maximum number of teams to compete against in the same weight is 11 [4].

It is important to know the demands of an activity and limiting performance factors of TOW pullers during current competition [6]. The TOW competition is characterized by active periods and rest periods. Among the latter are the rests between pulls against the same team and the rests between two matches. Periods of effort and recovery alternate repeatedly in which players require physical qualities such as muscular strength, power and muscular endurance in order to perform successfully. Related to performance, and strength in particular, during either dynamic or static pulling in a TOW competition, the maximal pulling force on the rope may be higher than 150% of the participants’ body mass [7], and regarding relative strength, the dynamic leg power can be 4659.8 ± 151.6 N. In this sense, given that strength is a key attribute of TOW, high levels of grip, back and leg strength are essential in order to resist the large forces generated by the opposing team [3]. Muscular contraction is primarily of the sustained isometric type as the participants resist the pull from the opposing team, alternating slow concentric and eccentric contraction against a heavy resistance [3]. In this sense, the intensity and effort response are greater and maintained above the anaerobic threshold during the competition in TOW pullers [6].

Given the particular nature of the sport [3], the duration of each pull and the sum thereof have implications for coaches and may indicate the need for greater consideration of the demands of the sport, with a view to maximizing the specificity and effectiveness of training programmers. Only two works have described the duration pulls in the scientific literature: one of them had a mean time of 2 min 30 s, but pulls lasting as long as 45–46 min have been recorded [3]; the second describes outdoor matches with a mean of 228.45 ± 129.95, with a minimum duration of 38 s and a maximum of 420 s [6]. The physical demands that may be required for pulls of less than 30 s or pulls of more than 10 min differ greatly and should condition the way that pullers train, whether in a more explosive or more aerobic/oxidative manner [8]. 

Describing data on each modality is essential in relation to the determining parameters for TOW performance: in particular, the duration of the competition, since each pull requires a maximum or almost maximum effort from start to finish; the cumulative volume of pulls (sum of total seconds pulling); and finally, the interaction with energy systems [9]. This would help in the preliminary diagnosis for decision making regarding program design, establishing training objectives, applying specific tests and any other evaluation in sports planning [10]. However, to the best of the authors’ knowledge, and based on a recent work published by our research group [11], in which the state of the art has been compiled, we have identified that no previous articles have described the duration of the indoor pulls, and even fewer data have been obtained during a follow-up on the best TOW athletes in the world during the highest level of competition. Therefore, the main aim of this article was to analyze the durations of the matches, differentiating between the first (T1) and second pull (T2) of each match. The pulls were compared in the qualification point phase and the final phase, as well as in each weight category that was competed. In addition, we have analyzed whether there are differences in the four World Championships considered. Finally, the total volume of pulls in each weight category was studied, both in the open club and in the national team competition. 

## 2. Material and Methods

### 2.1. Participants

A total of 479 TOW teams who participated in four World Indoor Championships: —Cesenatico, 2010; Pert, 2012; Castlebar, 2014; Volendam, 2016—took part in this descriptive follow-up study (Table 1).

The number of participant teams ranged from 119 teams in 2010 to 134 in 2016, with 113 team participants in 2012 and 2014. Considering the number of teams per weight, it can observe that the 600 k category presents the highest number (120 teams). In this investigation, we studied 3724 pulls. 

### 2.2. Procedure

The TWIF regulates global practice of the game [3] and has sponsored a World Championship event every 2 years since 1964 [1] for national and club teams. For the first 2 days of the World Championships, the club competition is held and is known as “open”, given that several teams of each National Association participate. For the other two days, national team competitions are played [4]. Furthermore, these TOW competitions involve weight classes, so weighting and stamping are necessary to complete the procedure (all weights). 

The duration of the pulls was registered by the official timekeeper/recorders of each Championships under the chief timekeeper/recorder’s supervision (each team uses their own chronometer system). These data were published and made available in the World Championships Official Results book and on the TOW International Federation website [4] a few days after the competition. In this study, the data of the four indoor TOW World Championships were selected on the following days: 2010 Cesenatico: 16 March; 2010/2012 Perth: 27 February; 2012/2014 Castlebar: 23 October; 2014/2016 Volendam: 23 February 2016.

In this investigation, we have taken into account the match duration of all senior categories except the mixed category (four women and four men), since this category was officially introduced for the first time in the World Indoor Championships in 2012 (Perth, Scotland). We have studied both the open club competition and the national team competition. The weights studied were as follows: women: 500 k and 540 k/men: 560 k, 600 k, 640 k and 680 k.

The duration of each pull was analyzed. Knowing that the competition is held over two or three pulls (semi-final and final), the first pull (T1) and the second (T2) were differentiated. The third pull was not taken into account, given that it represents less than 3.5% of the total number of pulls (18). The T1 and T2 were compared in the qualifying point phase and in the final phase in each of the weight categories. On the other hand, in order to obtain the maximum volume, each active team was added in the corresponding weights. 

### 2.3. Instruments

As mentioned above, all the data studied were obtained from the official TWIF website (http://tugofwar-twif.org/results/ (accessed on 10 November 2021)), so the instruments used were only those necessary to perform the statistical analyses, as be described in the following section.

### 2.4. Statistical Analysis

Data are presented as mean ± SD. All variables were tested with the Kolmogorov–Smirnov test (*n* > 50) to check the normality. The results were compared using Student’s t-tests for independent or paired variables as appropriate, or their corresponding non-parametric Mann–Whitney or Wilcoxon tests, in the case that the normality assumption was not met [12]. Cohen’s d for each dependent variable was calculated. A Cohen’s d of 0.2 was considered as a small effect, 0.5 as a moderate effect and 0.8 as a large effect [13] in the case of parametric tests and the rank biserial correlation; in the case of nonparametric tests, we considered <0.1 as a trivial effect, 0.1 as a small effect, 0.3 as a medium effect and 0.5 as a large effect. For the comparison of more than two groups, the one-factor ANOVA test was applied. In all cases in which different groups were compared, homogeneity of variances was taken into account through Levene’s test. Later, after the ANOVA, we used Tukey’s post-hoc analysis and the Games–Howell test depending on the result. Eta squared (η^2^) values are provided as a measure of effect size. An eta-squared effect size of η^2^ = 0.01 was considered to show a small effect size, an effect size of η^2^ = 0.06 was considered to show a medium effect size, and η^2^ = 0.14 was considered to show a large effect size [13]. The data were analyzed using the SPSS 21.0 software^®^ (SPSS, Chicago, Illinois, USA). The statistical difference was set up at *p* < 0.05.

## 3. Results

The descriptive data are shown in Table 2. A total of 1862 matches were registered. The first pull (T1) presented a time of 76.90 ± 51.75 s, and the time of the second pull (T2) was 60.96 ± 39.29 s.

Besides this, we observed statistical differences between T1 and T2: 15.72 sg (T1 > T2); (t = 19.51; *p* < 0.001; Es = 0.452; small effect). 

**Figure 1:**
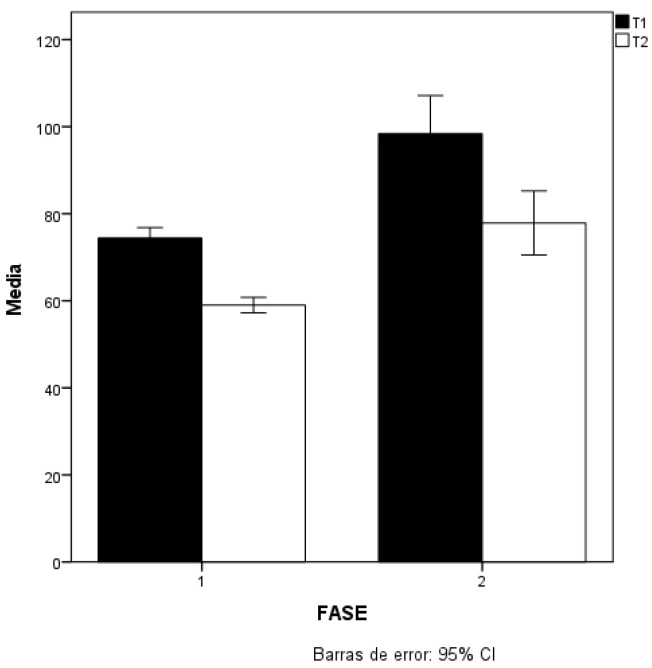
Comparisons of T1 and T2 in phase 1 and 2.

On the other hand, if we compare the matches in the two phases of the competition— the qualifying point competition (phase 1) and the final phase: semi-finals, finals and the matches for the bronze medal (phase 2)—differentiating between T1 and T2, the pulls were significantly longer, with *p* < 0.05 in both T1 and T2 (Table 3). T1 phase2 > T1 phase1; *p* < 0.05 (ES = 0.469; small effect) and T2 phase2 > T2 phase1; *p* < 0.05, (ES = 0.486; small effect) (Figure 1).

If we relate the durations of T1 and T2 in the two phases of competition for each weight category, the results showed significant differences for T1 between phase 1 and phase 2 in all weights except 500 k. In the second pull (T2), there were statistically significant differences for all the weights except for 640 k (Table 4).

The comparison among World Championships in T1 and T2 by gender showed some significant changes (Table 5). In particular, in the male group, there were not statistical differences according to the World Championships (year) (*p* > 0.05). However, in females, significant differences (*p* < 0.05) between the 2010 World Championships and the rest in T1 were observed (ES: 0.462 (small) for 2012, ES: 0.424 (small) for 2014 and ES: 0.680b (moderate) for 2016). In T2, there were significant differences between 2010 and 2014 (ES: 0.538 (moderate) and 2016 (ES: 0.895 (large), between 2016 and 2010 (ES: 0.895 (large); between 2016 and 2012 (ES: 0.48 small), and finally, between 2016 and 2014 (ES: 0.334 (small). The trend observed (although not significant in the rest of the cases) is that the length of the pulls has shortened over time. In both cases, the effect size is trivial (ES = 0.05 in T1 and ES = 0.094 in T2).

In order to determine the maximum volume of time in competition, the total number of seconds pulled by each club team and each national team were added for the weights that participated. It can be seen that in the female category, (500 k and 540 k), the maximum volume was 1996 s, while in the male category (560 k, 600 k, 640 k and 680 k), this corresponds to 2669 s, with 11 teams in the first case and 10 (two groups of 10) in the second (Table 6). In the same table, it can be observed that the competition volume is lower in the competition for national teams (WC) than in clubs (open).

## 4. Discussion

The main objective of this research was to analyze the durations of the matches, differentiating between the first (T1) and second pull (T2) of each match. The pulls were compared in the qualification point phase and the final phase, as well as in each weight category. In addition, any differences between the statistics for each of the four World Championships studied were analyzed. Finally, the total volume of pulls in each weight category was explored, both in the open club competition and in the national team competition. The main results are as follows: (1) the second pull is shorter than the first T1 < T2; *p* < 0.001, (Es = 0.452; small effect); (2) T1 and T2 times were longer in the final phase than the qualifying phase: T1 phase2 > T1 phase1, *p* < 0.05 (ES = 0.469; small effect) and T2 phase2 > T2 phase1, *p* < 0.05, (ES = 0.486; small effect); (3) there are statistical differences (*p* < 0.005) in T1 and T2 at all weights, except for T1 at 500 k and T2 at 640 k; (4) the comparison among World Championships does not present significant changes in the duration of the pulls; (5) however, in females, significant differences (*p* < 0.05) between the 2010 World Championships and the rest are observed in T1; (6) the volumes that we should take into account when designing the preparation should be those obtained when 11 teams compete, considering the maximum volume that we could find in the competition.

The pull is the main technical action that takes place in the TOW, where teams of eight pullers participate and apply enormous contra-directional forces on the pulling rope [2]. The pull duration values obtained in our study are lower than those found in the scientific literature [3,6]. These differences may be due to general and historical data [3] or to the specificity of the modality, since the data correspond to outdoor competition, and the difference in the shooting surface causes differences in friction and the way of supporting the feet. While in the indoor mode, the entire sole is supported to obtain the greatest friction between the materials (shoe sole and pulling surface) [14], outdoors, a greater grip is sought by participants drilling holes with their boots and using the same heels. One potential explanation is that the physical demands that may be required for pulling are very different and should condition the way that pullers train, whether more explosive or more aerobic/oxidative [8].

The results obtained regarding the duration of the matches reveal that the second pulls are shorter; it could be thought that this is due to the accumulation of fatigue. However, when comparing T1 and T2 in the qualifying point phase and in the final phase, both the first and second pulls are longer in the final phase, obtaining statistically significant differences (*p* < 0.05), as they accumulate more matches and presumably more fatigue [15]. This may be due to the fact that in the final phase, the teams are more equal and the level is more even, producing longer pulls. Therefore, the average duration of those pulls may be longer than in the qualification phase, where, due to the differences in levels, there are quite short pulls. In this sense, the coaches should take into account the duration of the pulls and establish training strategies that take into consideration the different metabolic pathways, according to the team objectives.

Furthermore, the competitions are based on weight categories; therefore, we compared the duration of T1 and T2 in the two phases of competition for each weight category (500, 540, 560, 600, 640 and 680 k). The results of the ANOVA test (one-factor) showed significant differences for T1 between phase 1 and phase 2 in all weights, except in 500 k. In the second pull (T2), there are statistically significant differences for all the weights except for 640 k. However, to the best of the authors’ knowledge, no previous data have been published in the scientific literature to explain these results. Therefore, future research is required in this field. Considering that, in most cases, the differences are significant, it does not seem necessary to follow a different strategy in the different weights with respect to the longer duration of the pulls in the final phase.

The comparison among World Championships in T1 and T2 by gender showed some interesting changes. In particular, in the male group, there are no differences according to the World Championships (year). The fact that a period of 6 years of follow-up in a World Championships does not present significant changes among competitions every 2 years (in the duration of the matches) reflects the homogenization of the tests to the maximum international level. However, in females, significant differences (*p* < 0.05) were found between the 2010 World Championships and the rest in T1. In this case, there is no previous scientific evidence to discuss this to the best of our knowledge. This significant difference could be because the number of female teams was lower than other years in 2010 (18 teams in 2010, 25 in 2012, 28 in 2014 and 34 in 2012). Based on fewer matches, the duration of the pulls could be longer than the rest of the years, where a long day of competition leads to greater muscle fatigue [16]. On the other hand, the results of T2 indicate that there are significant differences between 2010 and 2014 and 2016, between 2012 and 2016, between 2014 and 2016, and finally between 2016 and all other years. The trend observed (although not significant in the rest of the cases) is that the length of the pulls has shortened over time. In both cases, the effect size is trivial (ES = 0.05 in T1 and 0.094 in T2). The differences in the results between 2016 and the rest of the years may also be due to the greater number of female teams participating. However, we found no other scientific evidence that could explain the results. Therefore, future studies should analyze more female teams during championships.

In relation to the maximum volume of seconds in the competition, the number of teams clearly conditions this data—the more teams compete, the more pulls are made. However, the maximum number of teams with which to compete corresponds to 11, since if this number is exceeded, the teams will be distributed by groups [4]; therefore, the volumes that we should take into account when designing the preparation should be those obtained when 11 teams compete, considering the maximum volume that we could find in the competition.

## 5. Limitations

The main limitation of this descriptive study is that we do not know other international articles with which to compare the main characteristics of TOW.

## 6. Strengths

In this research, 1862 matches of 4 World Indoor TOW Championships have been studied. Besides this, 438 teams at the highest international level have participated, registering a total of 1862 matches and 3724 pulls, representing the peak of the elite level.

## 7. Practical Applications

These data may be used by coaches in order to understand better the times of the pulls; therefore, the following practical applications are proposed:

- The knowledge about the times of the matches helps us to design adequate training programs;
- Related to the total time of competition, these results could help strength and conditioning coaches to improve their sport planning and training;
- Finally, this article also helps to individualize training between men and women, considering the differences between the two genders.

## 8. Conclusions

The main conclusions are as follows: (1) the second pull is shorter than first, T1 < T2; (2) T1and T2 times were longer in the final phase than the qualifying phase; (3) there are statistical differences in T1 and T2 at all weights, except for T1 at 500 kg and T2 at 640 kg; (4) the comparison among World Championships does not present significant changes in the duration of the pulls; (5) however, in females, significant differences between the 2010 World Championships and the rest are observed in T1; (6) the volumes that we should take into account when designing the preparation should be those obtained when 11 teams compete, considering the maximum volume that we could find in the competition.

This first factor of the reality of TOW indoor competition, specifically the duration of the runs, allows a better understanding of the demands of the sport and the type of effort, aiding coaches in the design of training plans, maximizing their specificity and their effectiveness.

## Figures and Tables

**Table 1:** Number of teams and weight characteristics of the participants in four World Championships.

Year	2010	2012	2014	2016	4 Champs.
Weight	No Teams	No Teams	No Teams	No Teams	No Teams
500	9	11	13	12	45
540	11	14	15	22	69
560	27	28	19	21	95
600	32	25	29	35	120
640	24	20	21	30	94
680	16	15	16	14	60
no teams	119	113	113	134	479
no of pulls	992	864	864	1004	3724

**Table 2:** Descriptive analysis of T1 and T2.

Descriptive Statistical					
	N	Minimum	Maximum	Medium	Standard Deviation
T1	1862	6	309	76.90	51.78
T2	1862	7	362	60.96	39.29
N valid	1862				

**Table 3:** Analysis of T1 and T2 in phase 1 and 2.

	Phase	N	Medium	Standard Deviation	t	Effect Size
T1	1	1671	74.43	50.02		
	2	191	98.46	61.10	−5.238	0.469 (small)
T2	1	1671	59.02	37.17		
	2	191	77.90	51.51	−4.922	0.486 (small)

**Table 4:** Results of comparative analysis of pull durations (T1 and T2) in phase 1 and phase 2 by weight.

Gender	Weight		Phase	N	Average	Standard Deviation	t	*p*	Effect Size	
	500 K	T1	1	110	82.21	43.68	−1.775	NS		
			2	32	103.47	63.52				
W		T2	1	110	62.09	30.26	−2.065	*p* < 0.05	0.5	Moderate
O			2	32	78.84	42.88				
M	540 K	T1	1	224	61.23	41.67	−2.756	*p* < 0.01	0.749	Moderate
E			2	35	96.66	74.24				
N		T2	1	224	49.50	28.26	−2.159	*p* < 0.05	0.646	Moderate
			2	35	71.49	59.18				
	560 K	T1	1	337	95.99	57.82	−2.498	*p* < 0.05	0.462	Small
			2	32	122.72	57.94				
		T2	1	337	75.93	49.17	−2.497	*p* < 0.05	0.462	Small
			2	32	98.94	56.28				
	600 K	T1	1	478	75.96	51.46	−2.718	*p* < 0.01	0.496	Small
			2	32	101.97	65.20				
M		T2	1	478	60.47	36.25	−2.738	*p* < 0.05	0.798	Moderate
E			2	32	90.69	61.72				
N	640 K	T1	1	338	65.42	43.30	−2.522	*p* < 0.05	0.466	Small
			2	32	85.78	47.36				
		T2	1	338	51.21	28.71	−1.765	NS		
			2	32	60.81	36.10				
	680 K	T1	1	184	58.91	39.84	−2.288	*p* < 0.05	0.464	Small
			2	28	77.71	44.73				
		T2	1	184	48.38	28.89	−2.374	*p* < 0.05	0.577	Moderate
			2	28	65.71	36.97				

**Table 5:** Descriptive comparison among World Championships in T1 and T2 by gender.

GENDER			N	Average	Standard Deviation	95% Confidence Interval	
						Lower Limit	Upper Limit
	T1	2010	59	97.83	52.64	84.11	111.55
		2012	82	75.4	40.96	66.4	84.4
W		2014	103	75.5	43.64	66.97	84.02
O		2016	157	62.04	53.87	53.55	70.54
M		Total	401	73.5	50.00	68.59	78.41
E	T2	2010	59	77.39	34.96	68.28	86.5
N		2012	82	63.15	35.13	55.43	70.87
		2014	103	57.87	35.19	51	64.75
		2016	157	46.1	30.86	41.24	50.97
		Total	401	57.21	35.07	53.77	60.66
	T1	2010	437	78.24	57.31	72.86	83.63
		2012	350	81.07	51.45	75.66	86.48
		2014	329	76.75	50.4	71.28	82.22
M		2016	345	75.08	47.80	70.02	80.14
E		Total	1461	77.84	52.24	75.16	80.52
N	T2	2010	437	64.08	42.02	60.12	68.03
		2012	350	64.91	40.16	60.69	69.14
		2014	329	60,03	40.06	55.68	64.37
		2016	345	58.22	38.26	54.17	62.27
		Total	1461	61.98	40.32	59.91	64.05

**Table 6:** Maximum accumulative pulling time.

		2010		2012		2014		2016	
Weight	Comp.	No Team	Time	No Team	Time	No Team	Time	No Team	Time
500 K	Open	5	1387	7	1782	7	1321	5	1868 **
	WC	4	959	4	788	6	1196	7	1213
540 K	Club	7	1876	7	1370	9	1587	11	1996 **
	WC	4	1232	7	978	6	1225	11	1392
560 K	Open	17	2593	20	2669 **	12	1963	14	1688
	WC	10	2250	8	1733	7	1641	7	1659
600 K	Open	22	2155 **	18	1776	19	1941	25	1205
	WC	10	2114	7	1547	10	1872	10	1476
640 K	Open	16	1418	14	1362	14	998	21	2035 **
	WC	8	1689	6	882	7	952	9	1152
680 K	Open	9	1295	10	1385	9	1017	12	1062
	WC	7	1741 **	5	1060	7	1125	8	1155

** Maximum accumulated time in each weight.

## Data Availability

The data presented in this study are available within the article on the internet.
